# Why does the spread of COVID-19 vary greatly in different countries? Revealing the efficacy of face masks in epidemic prevention

**DOI:** 10.1017/S0950268821000108

**Published:** 2021-01-14

**Authors:** Jincheng Wei, Shurui Guo, Enshen Long, Li Zhang, Bizhen Shu, Lei Guo

**Affiliations:** 1 MOE Key Laboratory of Deep Earth Science and Engineering, College of Architecture and Environment, Sichuan University, Chengdu, China; 2 Department of Solid Waste Treatment Technology, Sichuan Environmental Protection Key Laboratory of Pollution Control for Heavy Metals, Sichuan Academy of Environmental Sciences, Chengdu, China; 3 Key Laboratory of Birth Defects and Related Diseases of Women and Children, West China Second Hospital of Sichuan University, Chengdu, China

**Keywords:** COVID-19, filtration efficiency, mask, public health, spread risk

## Abstract

The severe acute respiratory syndrome-coronavirus-2 (SARS-CoV-2) is highly contagious, and the coronavirus disease 2019 (COVID-19) pandemic caused by it has forced many countries to adopt ‘lockdown’ measures to prevent the spread of the epidemic through social isolation of citizens. Some countries proposed universal mask wearing as a protection measure of public health to strengthen national prevention efforts and to limit the wider spread of the epidemic. In order to reveal the epidemic prevention efficacy of masks, this paper systematically evaluates the experimental studies of various masks and filter materials, summarises the general characteristics of the filtration efficiency of isolation masks with particle size, and reveals the actual efficacy of masks by combining the volume distribution characteristics of human exhaled droplets with different particle sizes and the SARS-CoV-2 virus load of nasopharynx and throat swabs from patients. The existing measured data show that the filtration efficiency of all kinds of masks for large particles and extra-large droplets is close to 100%. From the perspective of filtering the total number of pathogens discharged in the environment and protecting vulnerable individuals from breathing live viruses, the mask has a higher protective effect. If considering the weighted average filtration efficiency with different particle sizes, the filtration efficiencies of the N95 mask and the ordinary mask are 99.4% and 98.5%, respectively. The mask can avoid releasing active viruses to the environment from the source of infection, thus maximising the protection of vulnerable individuals by reducing the probability of inhaling a virus. Therefore, if the whole society strictly implements the policy of publicly wearing masks, the risk of large-scale spread of the epidemic can be greatly reduced. Compared with the overall cost of social isolation, limited personal freedoms and forced suspension of economic activities, the inconvenience for citizens caused by wearing masks is perfectly acceptable.

## Introduction

Compared with the severe acute respiratory syndrome (SARS) coronavirus, Middle-East respiratory syndrome (MERS) coronavirus and influenza virus, the severe acute respiratory syndrome-coronavirus-2 (SARS-CoV-2) has a higher infectivity. As of 22 November 2020, 57 882 183 comfirmed cases and 1 377 395 death were reported worldwide [1]. Health care workers who have direct contact with patients must wear professional isolation masks to reduce the risk of infection in clinical settings. However, there is a debate as to whether the public should wear face masks in public areas or crowded rooms. The public epidemic prevention guidelines published by the World Health Organization state that only those who are directly exposed to patients need to wear protective masks [2]. Due to the differences in technology, culture and society background, there are great differences in understanding the use of masks in different countries. At the beginning of the epidemic, Japanese experts believed that the filtering function of masks against infectious viruses was limited [3]. Singapore only required people with respiratory symptoms to wear masks [4]. British and German experts pointed out that there was little evidence that masks can effectively prevent community respiratory tract infections, so it was not necessary to encourage people to wear masks [5, 6]. In contrast, the Chinese mainland, Hong Kong, Taiwan, South Korea and Thailand adopted widespread measures for the public to wear masks after the outbreaks of coronavirus disease 2019 (COVID-19). Although these Asian regions were first seriously affected by SARS-CoV-2, the establishment of a mask response mechanism in the COVID-19 epidemic played an important role in the prevention [7–9].

After the Asian pandemic, the COVID-19 spread more rapidly in Europe and the US, but was gradually brought under control in Asia, due to the implementation of strict public protection measures in the region. For example, the Taiwanese Center for Disease Control requires people to wear masks in public places, including public transport, and that will be fined if they fail to comply, and until now Taiwan has maintained good control of the epidemic without a mandatory suspension of work and school [10, 11]. Can masks play an effective role in the prevention and control of the spread of COVID-19? In fact, studies on the airborne characteristics of virus droplets provide some theoretical basis for explaining the protective efficacy of masks [12–15]. Study on the route of transmission of the SARS-CoV-2 shows that the airborne transmission route is highly virulent and dominant for the spread of COVID-19 [16]. From the perspective of transmission mechanism, as a respiratory virus, SARS-CoV-2 can cause upper respiratory tract infection through droplets, or it may be contained in aerosols and survive in the air for several hours [17]. Wearing a mask can filter out droplets within a certain range of particle sizes in the air, which has been proven by experimental studies of N95 respirator and surgical masks [18]. Reviewing the physical interventions used to contain respiratory transmission during past pandemics, it is found that wearing masks in groups can curb the spread of the virus to respiratory areas near individuals through exhaled droplets and air, thereby reducing the risk of transmission [19–23]. Studies on health care workers have shown that strict implementation of infection prevention by masks wearing is highly effective in reducing the risk of cross-infection in hospitals, both during the SARS in 2004 and the COVID-19 epidemic [24–27].

With the in-depth investigation of the epidemic, there is continuous evidence that asymptomatic people with SARS-Cov-2 are another major cause of virus transmission. Because they carry the virus, with a high concealment, it makes it more difficult to prevent and control the epidemic. The shedding of the virus from the asymptomatic carriers poses a great risk of transmission to the vulnerable population [10, 28]. The results of the ‘Diamond Princess’ cruise liner incident showed that 18% of all infected individuals with SARS-CoV-2 in nasopharyngeal and oropharyngeal swabs did not show any symptoms [29]. In the clustered virus transmission that occurred in Singapore from 23 January to 16 March 2020, it was estimated that more than 6% of population infections were caused by asymptomatic carriers [30]. In addition, the results of quantitative virus detection showed that the viral loads in nasopharynx and oropharynx samples in asymptomatic and pre-symptomatic individuals of COVID-19 patients were similar to those of symptomatic individuals, and a large number of virus loads were found in the upper respiratory tract of patients, indicating a great risk of infection [31, 32]. This is very different from the situation in regard to SARS patients in that the virus mainly exists in the lower respiratory tract [33, 34], and the amount of viruses contained in asymptomatic individuals of influenza patients is much lower than that of symptomatic individuals [35, 36]. Some scholars have used model studies to show that if there is no intervention, asymptomatic infection may become the main culprit leading to the large-scale spread of COVID-19 [37]. Therefore, for people who are vulnerable to infection, it is very important to use masks as a self-protective measure when they cannot accurately identify whether there are asymptomatic patients around them. Some Asian national health institutions with more stringent public prevention and control measures have proposed that a key factor in requiring the public to generally wear masks is the high risk of asymptomatic infection [31]. The latest model simulation studies show that masks are effective in preventing healthy people from being infected by asymptomatic infections, and even home-made masks can reduce the risk of community infection [38, 39]. A study on the effect of face masks on the spread of COVID-19 in Germany using the synthetic control method indicate that the early introduction of face masks in Jena has resulted in a drop in newly registered COVID-19 cases of around 75% after 20 days [40]. Some countries and regions strictly implement public policy to wear mask to loosen the lockdown interventions and to promote the resumption of normal life, and so far the epidemic has not broken out again, providing a stable guarantee for a return to normality [10]. Therefore, compared with the initial stage of the epidemic, European and American countries have gradually begun to change their views on wearing masks. The Center for Disease Control and Prevention (CDC) advised the public to use cloth masks on 3 April 2020, especially for people in areas with high risk of community outbreaks [41]. Western scholars have re-emphasised the importance of adopting a public mask policy, and suggestions to encourage children to wear masks have been repeatedly put forward [10, 42]. The WHO also updated its guidelines on the use of masks on 5 June 2020, suggesting that governments should encourage the public to wear masks in situations where the epidemic is widespread and physical distancing is difficult to maintain, such as on public transport, shops or other crowded environments.

However, there is still no conclusion about the role of masks in the prevention and control of COVID-19 epidemic, and whether the use of masks can effectively protect healthy people from infection and greatly reduce the risk of transmission. Some scholars have questioned on the basis of the mechanism of droplet and air transmission of the virus, it is considered that masks can only filter large particles exhaled by patients, and ordinary masks other than N95 masks and surgical masks (such as cotton masks and home-made masks) cannot provide effective protection for healthy or vulnerable people [8, 37]. But based on the transmission route of infectious droplets, some scholars have proposed that those large droplets are the main conflict in disease transmission; thus, even if they are blocked by a home-made mask initially, it significantly reduces the risk of virus transmission [43]. In this paper, by integrating the experimental research of all kinds of masks, the filtration performance of different types and materials of masks is summarised in a quantitative way. Combined with the study of the particle-size distribution of patients' exhaled droplets and the results of virus load testing, the protective efficacy of masks can be explained scientifically, so that the public can clearly understand the role of masks in the prevention and control of epidemics. It has a positive effect on improving public health prevention and control measures, enhancing public protection, curbing the further deterioration due to the epidemic, and consequently promoting the resumption of work, and stabilising social order in areas with serious epidemics.

### Comparison of masks filtering effect based on experiments

As a personal protective equipment (PPE), the protective performance of mask is affected by many factors, such as material properties, air flow, surface air pressure, wearing method, facial fitting etc. There are many methods and indicators to evaluate the performance of masks. In terms of curbing the spread of respiratory virus transmission, whether it can effectively prevent toxic exhalation and effectively filter viral particles in the air to provide a strong virus isolation barrier for healthy people, is the key to evaluate the performance of the mask. Therefore, this section retrospectively reviews mask filtering experimental studies (Table 1) undertaken by scholars, doctors and research institutions around the world for various virus epidemics, comprehensively sorting the wide variety of research results, and objectively summarising the general performance of mask protection.

**Table 1.** Summary of mask studies

Author(s) (Year)	Key points	Type of Particles	Interventions	Performance evaluation
Lee SA *et al*. (2008) [47]	Laboratory volunteer experiment; 12 healthy volunteers; OSHA test	NaCl aerosol (virus substitute)	Four models of N95 respirators and three models of surgical masks	Protection factor
Johnson DF et al. (2009) [49]	Laboratory patient experiment; nine patients coughed with masks; the Petri dish receives coughing particles	Influenza virus	Without masks N95 respirators Surgical masks	Virus detection load: cycle number
Radonovich LJ *et al*. (2019) [25]	Random case−control study; 2862 medical staff in influenza clinic	Influenza virus	N95 respirators Surgical masks	Number of infected health care workers
John D. Noti *et al*. (2012) [50]	Laboratory model experiment; cough mannequin; Breathing mannequin (wearing a mask)	Influenza virus	N95 respirators Surgical masks	Virus detection load
Seongman Bae *et al*. (2020) [51]	Laboratory patient experiment; four COVID-19 patients; Petri dish receives cough virus	SARS-Cov-2	Surgical masks Cotton masks	Virus detection load
Milton DK *et al*. (2013) [48]	Laboratory patient experiment; 37 influenza patients; Collect exhaled particles from patients	Influenza virus	Surgical masks	Reduction of virus load by multiple
Tara Oberg *et al*. (2008) [45]	Laboratory infiltration experiment; aerosol particles of different sizes guided by air flow through the mask	Latex ball aerosol NaCl particles	Surgical masks Dental masks	Penetration rate
Derrick JL *et al*. (2006) [46]	Laboratory volunteer experiment; eight healthy volunteers; Tape sealing to improve the tightness of mask	Air dust particles	Surgical masks Laser masks FFP2	Reduction of virus load by multiple
J.L. Derrick *et al*. (2005) [52]	Laboratory volunteer experiment; six healthy volunteers; Collection of exhaled particulate matter	Human exhaled particles	1-layer, 2-layer, 3-layer and 5-layer surgical masks	Reduction of virus load by multiple
Angela Weber *et al*. (1993) [53]	Laboratory model experiment; Breathing mannequin (wearing a mask)	Corn aerosol	Eight surgical masks	Penetration rate
Anna Davies *et al*. [44]	Laboratory volunteer experiment; 21 healthy volunteers; Cough box to collect target particles	*B. atrophaeus* Bacteriophage MS2	Surgical masks Home-made masks	Filtration efficiency

The types of masks involved in these experimental studies are: N95 respirators, surgical masks, dental masks, laser masks, home-made/cotton masks, self-made new material masks and so on. The type of experiment is basically divided into laboratory experiments and patient experiments, and some scholars directly carried out on-the-spot detection and follow-up investigation of hospital medical staff during the epidemic. In the experiments, researchers generally adopted the case−control or random case−control approach. With the approval of the relevant health institutions, they recruited experimental volunteers or patients clinically diagnosed with a certain virus, and carried out experiments according to specific guidelines. Some models developed by researchers to simulate real human breathing have also been used in mask research experiments (perhaps for the actual protection of people). In volunteer experiments, people were generally asked to wear different types of masks, and then they were asked to cough, talk, breathe according to the guidelines to create airflow conditions for mask filtering. Then using a Petri dish or microbial sampler to collect target particles (viral aerosols, droplets or other exhaled particles) released in the laboratory. Finally, the sampling probe was used to collect the target particles at different measuring points and different levels of the mask. According to the number of target particles collected (through scientific studies, mathematical statistics and error analysis), the researchers were able to set various indicators to evaluate the filtration performance of masks, such as filtration efficiency [44], permeability [45, 46], protection factor [47], virus reduction multiple [46, 48] and the virus concentration before and after filtration [49–51] etc. In addition, some scholars carried out their research study in hospitals, and monitored and followed up with the medical staff who encountered a large number of patients every day, and divided them into experimental groups according to the actual wearing of masks. The protective efficacy of the mask was indicated by the actual infection data [25].

A systematic review of the experimental results of the masks included in Table 1 shows that the comprehensive performance of N95 masks in particulate filtration is better than that of other types of masks. The recommendations of National Institute for Occupational Safety and Health to wear masks for the public during the outbreak also focused on N95 respirators and surgical masks, with little mention of other types of masks. The virus filtering performance of cotton masks, home-made masks or other types of masks in the medical field (dental masks, disposable medical masks etc.) are considered to be poor. There is no doubt that there are differences in filtering performance among the different types of masks due to the inconsistency in the design and using. The N95 mask is designed to prevent the wearer from inhaling small particles in the air, meeting the filtration requirements, while the surgical mask is designed to prevent the spread of microbes from the wearer to other people. For other ‘questioned’ types of masks, how different are their actual protective effects compared with N95 and surgical masks, and what are the differences. We need to use scientific and quantitative data for in-depth analysis.

**Fig. 1.**
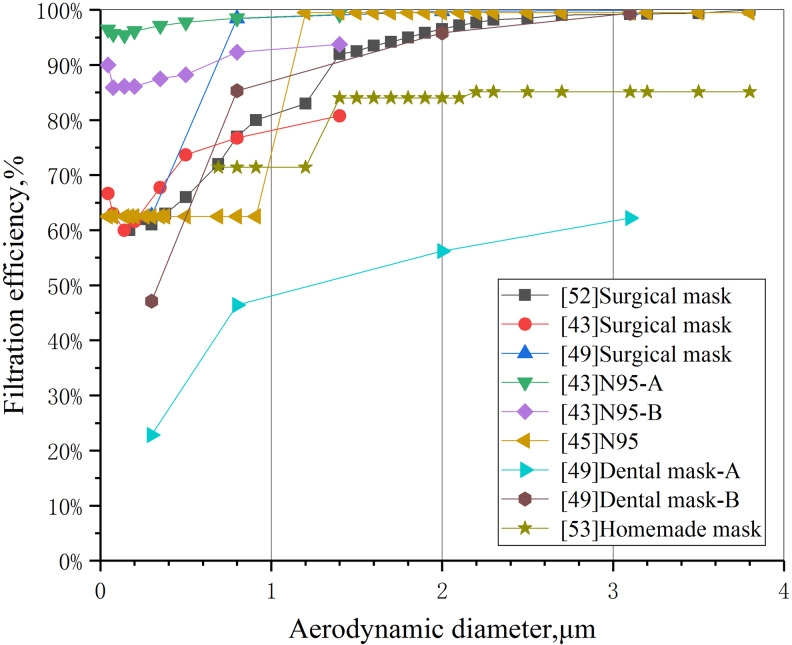
Comparison of filtration efficiency of several commonly used masks.

The filtration performance of masks varies greatly with different particle sizes, which is generally accepted in the field of mask experimental research, and it may also be the root cause of criticism of ordinary masks. As early as 1993, Angela Weber *et al*. from University of Cincinnati tested the permeability of surgical masks with aerosol particles of different sizes in a 2 m^3^ test chamber using a human respiratory model. The test results showed that the permeability of surgical mask varied clearly with the particle size, and the permeability was higher for particles less than 1 μm, but close to 0 for particles above 3 μm [53]. In an experiment in which the protection factor (PF) was used as the evaluation index of mask performance, Lee *et al*. compared the protection level of 12 subjects who took part in health tests of the Occupational Safety and Health Administration of the United States (OSHA) after wearing N95 filter masks and surgical masks. In the OSHA test, subjects were asked to breathe normally, breathe deeply, turn their head left and right, move up and down, talk, make faces, bend over and return to normal breathing under the condition of wearing a mask for 2 min. The average concentration of particulate matter in the mask was taken within 2 min. The experimental results showed that the N95 mask had a higher PF for larger particles above 1 μm [47]. More filtration experiments showed that cotton masks [51], dental masks [45], laser masks [46] and multi-material home-made masks [44] all showed different filtration levels in different particle sizes. In this paper, the results of these mask filtration experiments were analysed and integrated, and the filtration performance indicators used in different experiments were transformed into mask filtration efficiency, to obtain the distribution characteristics of masks' filtration performance in different particle-size ranges (Fig. 1).

Figure 1 shows that the variation of filtration efficiency with particle size of all kinds of masks is highly consistent. About 1 μm is the point of sudden change (threshold) in mask filtration efficiency. No matter what type of mask, when the particle size exceeds this threshold, the mask filtration efficiency will be significantly improved and stabilised at a higher level (the average efficiency is more than 80%). When the particle size is less than 1 μm, the filtration efficiency of most other types of masks is only 60%–70% (except for the N95 mask [47]). It should be pointed out that the international standard for evaluating the performance of masks is based on the filtration efficiency for 0.3 μm particle size [54], while the filtration efficiency of dental masks (Dentalmask-A) at 0.3 μm in [45] is less than 30%, which is the main reason why the protective efficacy of masks is questioned. Some scholars have pointed out that the filtration performance defect of masks in the range of small particle size has become a huge hidden danger in personal protective measures. Because there is no way to stop the small particles of viral droplets exhaled by patients, people cannot reduce the risk of infection even if they wear masks [48]. However, this view is one-sided, and the evidence is shown later.

**Fig. 2.**
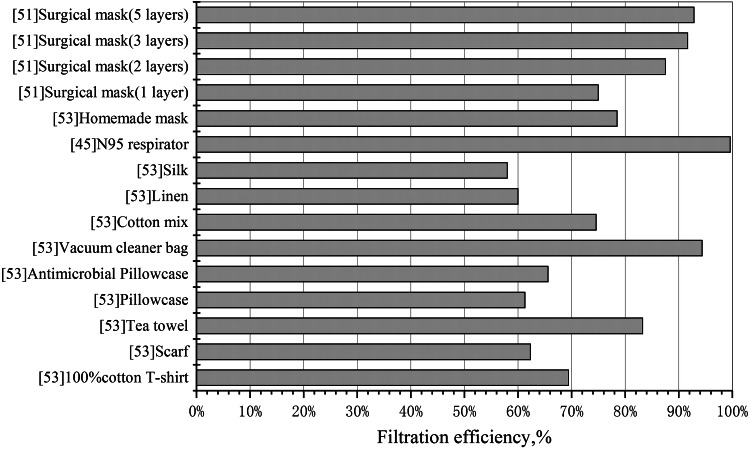
Comparison of AFE of 1–4 μm particles by common filter media and mask.

In addition, the data in Figure 1 show that the filtration efficiency of N95 masks and medical surgical masks is basically maintained at a high level in various particle-size ranges, especially for particles less than 1 μm. N95 is significantly better than surgical masks, and surgical masks are significantly better than other types of masks, which has greatly stimulated the demand for these two types of masks all over the world. In some areas where the production scale of masks is limited, the government restricts the release of masks to the public in order to ensure the demand for personal protective materials for health care workers is met, resulting in a shortage of N95 and surgical masks in society [7]. As a two-way solution, some suggestions have been put forward to encourage the public to use common materials to make masks for themselves in order to ease the pressure of production, but these suggestions are controversial [44]. For example, cotton masks are suspected to be used only to block visible particulate contaminants and have little filtering effect on aerosols or droplets [45]. Some members of the public also lack confidence in the antiviral effect of home-made masks and are prone to anxiety when the supply of N95 and surgical masks is insufficient. Whether the mask made by common materials can provide effective protective function needs to be proved by scientific experimental data. In this paper, the data of filtration experiments involving a variety of materials and masks are processed, and the average filtration efficiency (AFE) of common filter media and masks in the particle size of 1–4 μm is compared as shown in Figure 2.

The data in Figure 2 shows that in the range of 1–4 μm particle size, the AFE of common materials such as plastic, silk, linen and cotton cloth is more than 50%. Among them, the AFE of vacuum cleaner bag, tea towel and cotton mix is close to that of surgical mask and N95 respirator. The home-made mask in Figure 2 is a household mask made by volunteers using cotton *T*-shirts, with an average filtering efficiency of nearly 80%. Thus it can be seen that many common materials and masks can filter out most of the viral particles with a particle size of more than 1 μm, and it would be biased to completely deny the efficacy of using masks only because of the lack of filtering efficiency for particles with a size of less than 1 μm. At a time when there is an extreme shortage of N95 respirators and surgical masks, it is also a constructive suggestion to encourage the public to make protective masks using common materials such as cotton, vacuum bags and tea towels.

The distribution of the filtration efficiency of the mask with the particle size is only a single factor quantitative result obtained under laboratory conditions. To evaluate the actual protective efficacy of wearing a mask for the healthy public, it is also necessary to further identify the relationship between the particle-size distribution of droplets coughed (or exhaled) by patients and the viral load. The experiments of Davies *et al*. showed that the droplets coughed by influenza patients contained different amounts of virus in different particle sizes [44]. Based on the tests of cough droplets in several particle size less than 7 μm, the authors found that droplets of the size of 1.1–2.1 μm contain the largest number of influenza viruses. For patients with SARS-CoV-2, there are differences in the amount of viruses in cough droplets or viral aerosols of different sizes. Therefore, we need to reveal the relationship between droplet size distribution of coughed droplets and viral load of COVID-19 patients, in order to further demonstrate the actual protective efficacy of the mask.

## Particle-size distribution of droplets and viral load of patients

**Fig. 3.**
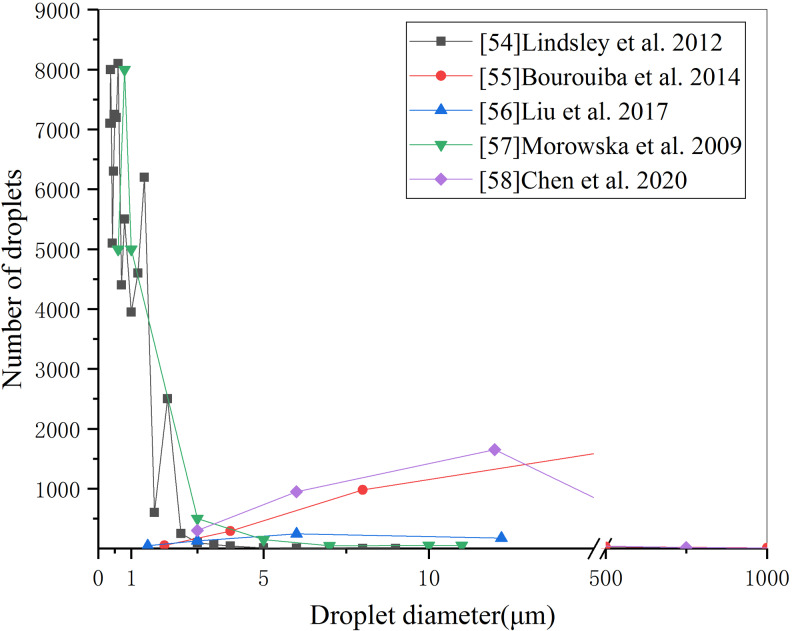
Distribution of exhaled droplets with particle size.

The above experimental evidence shows that the filtration efficiency of all kinds of masks varies with the particle size. Do the droplets exhaled by patients have different quantitative distributions in different particle sizes? In fact, the number of droplet particles exhaled by the human body (whether healthy or infected) due to normal breathing activities or behaviour such as speaking, coughing and sneezing also shows specific distribution characteristics with the change of particle size. In this paper, the measured data of particle-size distribution of one-time exhaled droplets of influenza patients coughing [55] and healthy volunteers sneezing, intense breathing [56] and cough [32, 57, 58] were extracted from the literature and presented in Figure 3. Whether infected or healthy people, the number and particle size of exhaled droplets show a lognormal distribution, but the results given by different scholars are very different due to different test methods and concerns. For example, the peak number of 0−5 μm droplets measured by intense respiratory activity [58] is about 4000–8000 and the number of particles in the size range of 5–75 μm is about 1000, while the number of large particles above 75 μm is very small. For patients with influenza, the number of droplets with a particle size of 2.5 μm was almost 200–1000 times larger than that of more than 20 μm [55].

**Fig. 4.**
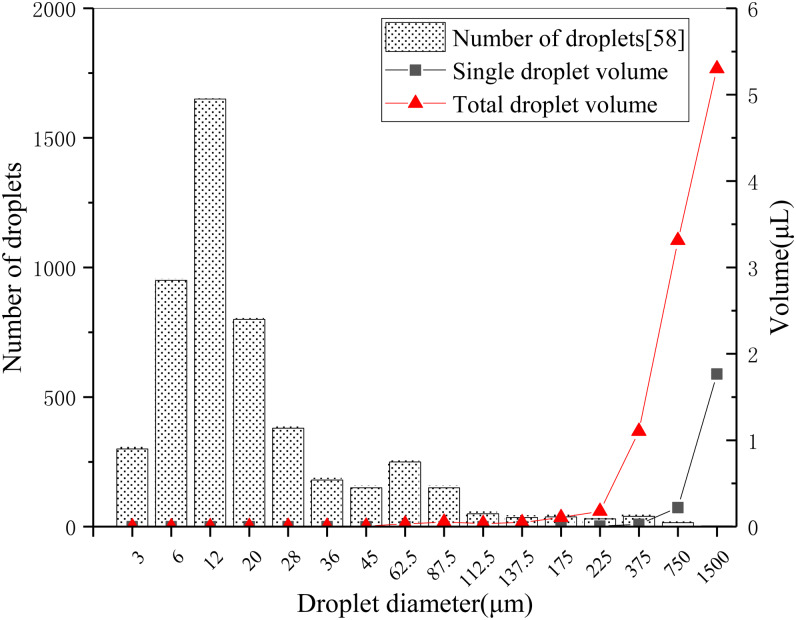
Distribution of exhaled droplet volume with particle size and number in a cough.

However, the volume of small droplet particles which are dominant in quantity is not necessarily larger than that of a small number of large droplets. Taking the quantity distribution of exhaled droplets of different sizes as an example [32], the volume of droplets with different sizes can be approximately calculated by using the sphere volume formula, and then the total volume distribution curves of droplets with different sizes can be obtained by considering the number of droplets with different diameters, as shown in Figure 4. Because of the cubic relationship between particle volume and particle size, the difference of particle size by 10 times will lead to a volume difference of 1000 times. From Figure 4, even if the number of cough exhaled particles below 50 μm is much higher than that above 100 μm, the total volume of a small number of particles above 100 μm is much larger than that below 50 μm. As can be seen from Figure 1, when the particle size exceeds 4 μm, the filtration efficiency of all kinds of masks basically reaches 90%. On the other hand, for larger particles (such as 10–1000 μm), the filtration efficiency of even ordinary self-made cotton masks will be close to 100%. Therefore, although the substandard ‘questionable’ mask does not have an excellent effect on small particle-size filtration, its filtration effect on large particle-size droplets is equivalent to that of N95 respirator and surgical mask. From the point of view of blocking the total volume of foam droplets discharged from breathing, coughing and sneezing of infected people, based on the filtration efficiency distribution curves of [47] N95A and [44] home-made mask in Figure 1 and the particle-size distribution curve of exhaled droplets of human intense respiratory activity in Figure 3 [58], the overall filtration efficiency of N95 and home-made masks on exhaled droplets within the full particle-size range is simulated and calculated, and the results are 99.4% and 98.5%, the difference is only 1%.

**Fig. 5.**
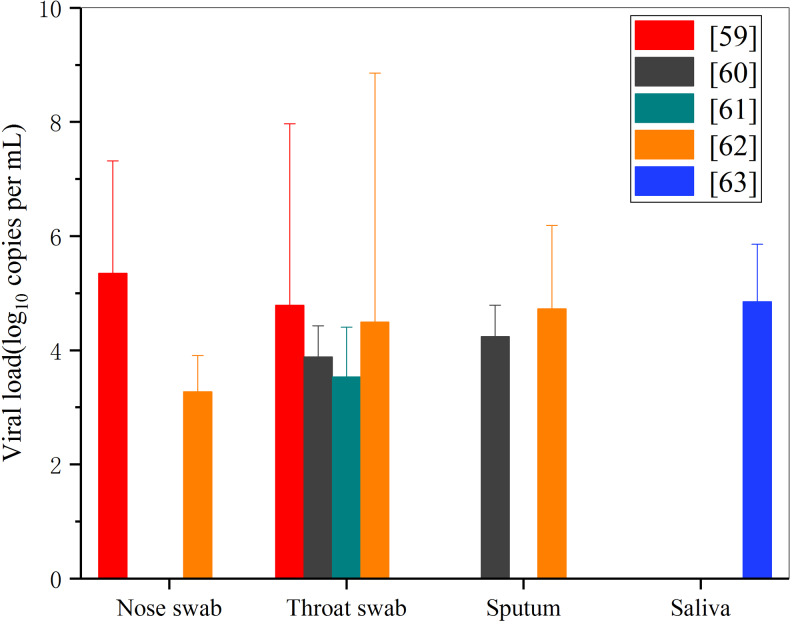
Comparison of virus load in COVID-19 patient test samples.

The main source of the transmission of respiratory diseases (including SARS-CoV-2) is that the droplets that can enter into the environment of droplets released by the host through the oropharynx, nasopharynx and throat. This paper analyses the viral load data of five different researchers on the concentration of virus in nose and throat swabs of patients with COVID-19 [59, –63]. Figure 5 shows the average level of viral load in nose, throat, sputum and saliva samples of COVID-19 patients from different literature sources within 5–8 days after infection and diagnosis (the peak period of viral load), and the peak value is also marked.

It can be seen that at 5–8 days after onset, the average viral load of nose swab, throat swab, sputum and saliva in COVID-19 patients is 10^3^–10^5^ copies/ml, and the highest viral load may be up to 10^8^ copies/ml ([62] suggests it can be as high as 10^11^ copies/ml). Although the amount of SARS-Cov-2 virus in the droplets exhaled from the mouth, nose and throat varies greatly due to individual differences, and the amount of upper respiratory tract virus in the same infection varies significantly in different periods, if the mask is worn, the shielding efficiency of the mask against droplets with different particle sizes will not change because of this individual difference. The viruses are evenly and randomly distributed in oropharynx, nasopharynx and sputum, with more viruses in large droplets. Therefore, the larger droplets of infected people in a breathing activity will contain a larger number of viruses, which will be more dangerous and have a greater risk of transmission. Wearing masks (whether N95, surgical masks or ordinary cotton masks) has similar high filtration performance for these large particles, which can greatly reduce the probability of excessive virus intake in healthy people who encounter infected patients.

The above conclusions can explain the results of some investigations and studies on the protective effect of masks. Johnson *et al*., experts at the Infectious Disease Research Institute in Heidelberg, Germany, confirmed through patient experiments that wearing N95 respirators can change the droplets in Petri dishes coughed by influenza patients from positive to negative [49]. A study on virus detection in four COVID-19 patients in South Korea showed that both ordinary cotton masks and surgical masks could greatly reduce the amount of SARS-Cov-2 virus coughed into the air. Compared with the amount of virus in saliva coughed without a mask, wearing a mask could reduce the amount of the virus by 10–100 times, and the protective effect was significant [51]. Therefore, it is one-sided to conclude that the traditional view of poor protective efficacy of masks is based on the experimental results of the high permeability of masks to single small particles, and people should eliminate misunderstandings about ordinary masks and home-made masks. In the face of an extreme shortage of N95 respirators and surgical masks, the public wearing of ordinary masks or home-made masks can still greatly reduce the risk of COVID-19 transmission.

## Discussion

Currently, the COVID-19 epidemic, which has caused the global pandemic, is spreading in more than 100 countries and regions, and may continue to threaten human public health and security for a long time. The spread of the epidemic is difficult to control, many countries around the world have been forced to take emergency measures to lock down cities and shut down commerce, and the economy has been greatly affected. As most countries are in a state of blockade, consumer demand for products and services has fallen sharply, bringing domestic and international production and service supply chains to a standstill, and the sales and liquidity of large international listed companies have been greatly affected. Coupled with the impact on human beings and health systems, the epidemic exacerbates the unemployment, which further leads to a decline in demand and a global economic recession [64]. Currently, gatherings and large-scale population movement around the world are considered as high-risk social behaviours, and all kinds of large-scale international events have had to be cancelled. Because of the epidemic, the 2020 Tokyo Olympic Games has become the first Olympic event that was postponed [65]. The changes in the global social order caused by the epidemic also have a great impact on people's mental health. European social surveys show that quarantine and travel restrictions have led to a significant increase in the prevalence of depression, anxiety or insomnia in the public, and the prevalence of severe mental symptoms in the UK is three times higher than that in Europe [66]. Research findings from Uganda shows that COVID-19 public health restrictions have a severe negative impact on the lives of older adults, affecting their basic existence and causing the inability for them to have access to sufficient food, healthcare and education for their grandchildren [67].

Under such a grim situation, the urgent task is to promote the restoration of global social order as soon as possible, to resume work and economic activities under the premise of ensuring safety, resuming international cooperation and exchanges, and to bring social development back to the normal track. It requires the adoption of global public health measures to provide comprehensive and strong health protection for society. Based on this situation, we believe that it is imperative to implement the policy of wearing masks for the public. On the one hand, wearing a mask is simple, practical and feasible self-protection behaviour for every individual, and it has no great resistance in technology or cost. On the other hand, this our study also confirms the protective effect of masks through actual data. Wearing masks can provide a strong guarantee for healthy people and can effectively block the vast majority of toxic droplets exhaled by infected people. Even ordinary masks and home-made masks can filter out the large droplets that account for most of the volume, thus blocking most viruses and greatly reducing the probability of infection. This conclusion can explain the model predictions that universal masking is of high value in reducing community transmission and in controlling pandemics. It is also consistent with the positive progress made in epidemic control in areas that have incorporated mask use policies into stringent public health prevention and control measures [38].

At present, scholars from all over the world support the use of masks more and more loudly, and people in Europe and the United States are paying increased attention to masking. Under the situation that the epidemic will exist for a long time, we believe that the traditional view of the uselessness and skepticism of mask use should be ignored, and there is no doubt that wearing masks can significantly reduce the risk of transmission compared with not taking any protective measures. In the areas where the epidemic is serious, the use of masks by the whole population is undoubtedly a strong public health protection measure. The combination of the individual wearing of masks with other protective measures and the active epidemic prevention of all members of society will certainly have a beneficial effect on the prevention and control of the epidemic. Based on Figure 1, the filtration efficiency of different masks for large particle size and extra-large droplets can reach nearly 100%, and according to the volume distribution of droplets of different particle sizes released by the infected people during breathing activities, as shown in Figure 4, we have reason to believe that if the policy of wearing masks by the whole society (even ordinary masks or self-made masks) is seriously implemented, after the normal production, commercial and social activities resume, the risk of spreading the epidemic can be reduced to a very low level. Compared with social isolation, the limitation of personal freedom, and the overall cost of economic losses caused by the cessation of industrial and commercial social activities, the inconvenience of wearing masks is negligible.

## Conclusions

The results of mask filtration experiments show that the filtration efficiency of all kinds of masks has similar distribution characteristics depending on the particle size, and the efficiency is higher for large particles. However, it is one-sided to question the epidemic prevention effect of wearing masks. This paper describes the characteristics of the mask filtration efficiency distribution with human exhaled droplets size distribution, integrates relevant research results, analyses the experimental data, and concludes that in the droplets exhaled by COVID-19 patients, although the number of small particles absolutely dominate, the volume proportion of large particles is much larger than that of small particles. Large particles contain more SARS-Cov-2 viruses, resulting in a greater risk of transmission. Therefore, N95 respirators, surgical masks, ordinary cotton masks and home-made masks can filter out the vast majority of viruses by blocking the large droplet particles, significantly reducing the risk of infection in healthy people. Universal masking will have a significant influence on the prevention and control of the epidemic. Additionally, the negative impact of wearing masks is far lower than that of social isolation restrictions and the cessation of industrial and commercial activities.

## Data Availability

The data used to support the findings of this study are available from the corresponding author upon reasonable request.
